# Supplementary material for: Cis-regulatory polymorphism at fiz ecdysone oxidase contributes to polygenic evolutionary response to malnutrition in Drosophila
Source: PLoS Genet. 2024 Mar 7;20(3):e1011204. doi: 10.1371/journal.pgen.1011204 (PMC10962836; doi:10.1371/journal.pgen.1011204)
Supplement: S1 Appendix — (PDF) [file pgen.1011204.s001.pdf]

Cis-regulatory polymorphism at *fiz* ecdysone oxidase contributes to polygenic adaptation to malnutrition in *Drosophila*

S1 Appendix

Fanny Cavigliasso, Mikhail Savitsky, Alexey Koval, Berra Erkosar, Loriane Savary, Hector Gallart-Ayala, Julijana Ivanisevic, Vladimir L. Katanaev, Tadeusz J. Kawecki

Supplementary Figures A-F

Supplementary Tables A-M

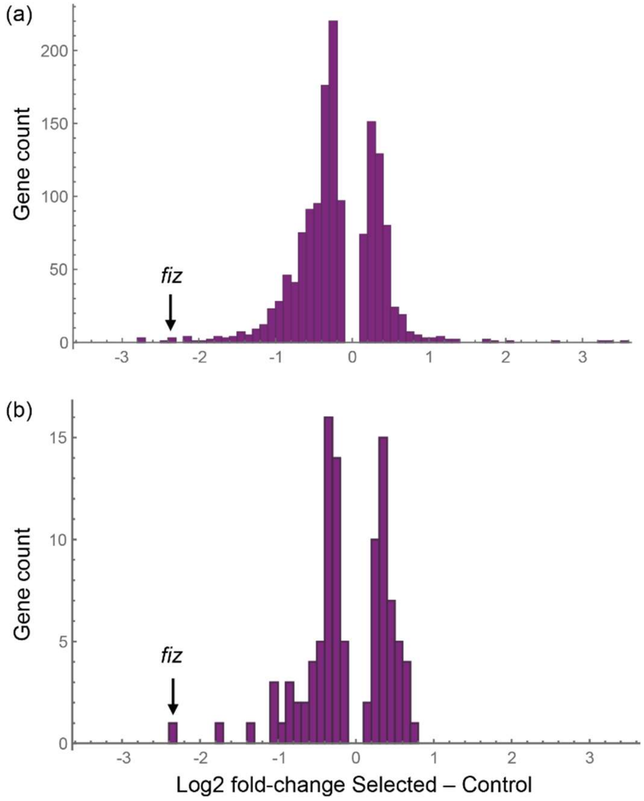

Supplementary Figure A. The distribution of estimated differences between the mean expression Selected and Control populations for (a) all 1464 genes differentially expressed (at 5% FDR) between the two sets of populations and (b) 102 differentially expressed genes with a least one candidate SNP within 5 kb of their gene boundaries as defined in FlyBase (flybase.org). Differentially expressed genes were identified by RNAseq on 3rd instar larvae raised on the poor diet (ref. 11); candidate SNPs were identified as those with significant divergence in allele frequencies between Selected and Control populations (also at 5% FDR) (ref. 18). The arrows indicate the position of *fiz* in the histograms.

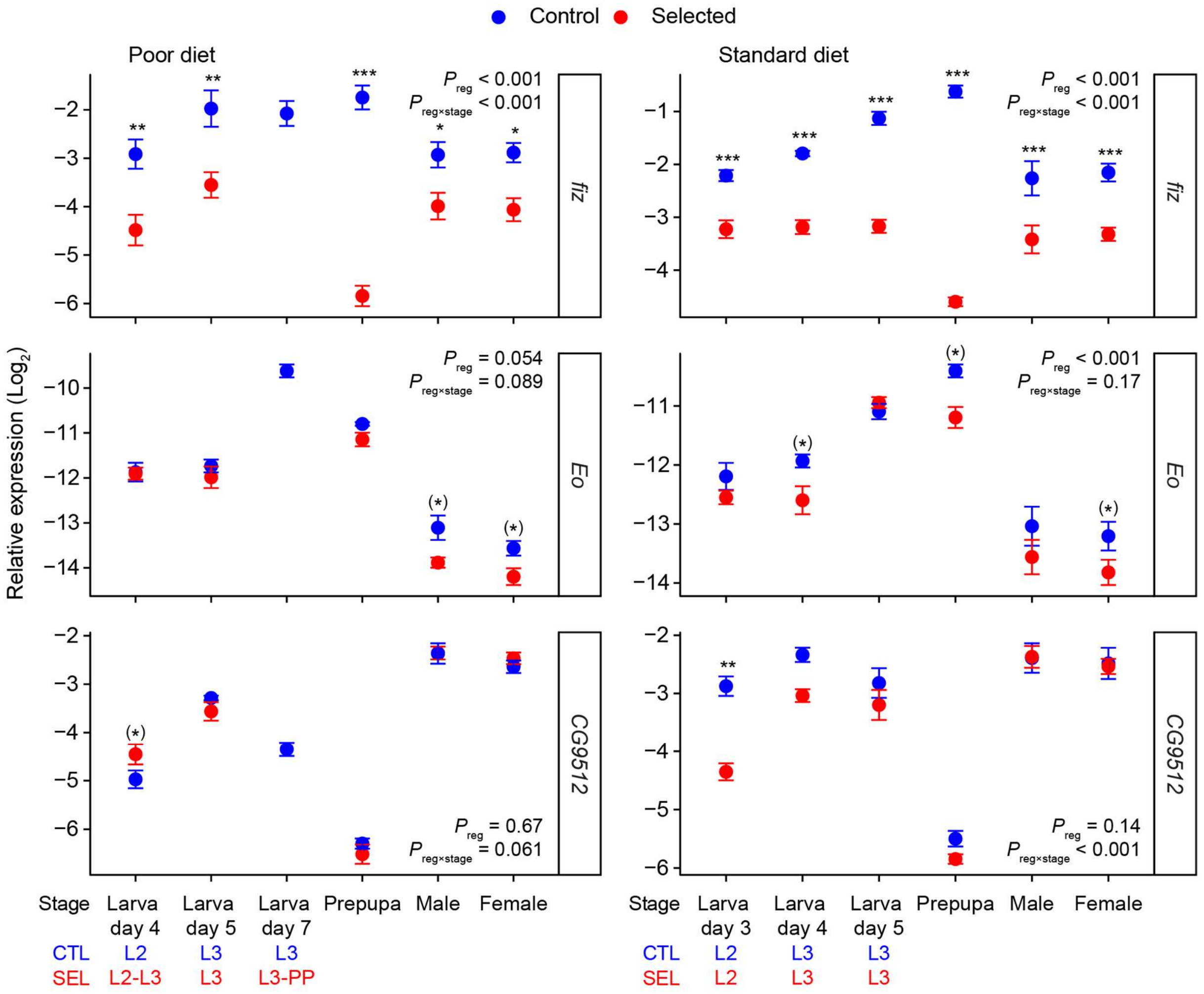

Supplementary Figure B. Relative expression (Log_2) of *fiz, Eo* and *CG9512* on both diets and across developmental stages. Larvae were collected on day 3 to 7 post egg laying. In an independent experiment, we checked the corresponding larval stages for Selected (SEL) and Control (CTL) populations (indicated below the X-axis). On day 4 on poor diet, there were only L2 for CTL whereas there were ¾ of L2 and ¼ of L3 for SEL. There are no data on day 7 for Selected larvae on poor diet because most of them pupariated by then. L2 and L3: second and third instar larvae. For each gene and diet, the significance of the main effect of the selection regime (P_{reg}) and of the regime × stage interaction ($P_{reg×stage}$) are shown. Asterisks indicate a significant difference between Control and Selected populations at the particular developmental stage after *p*-value correction (sequential Bonferroni adjustment for stages). (*) implies the $P < 0.05$ before but not after the correction. Symbols are means ± SE. N = 3 populations × 2 replicates per selection regime, diet and stage. Each replicate consisted of a pool of seven individuals (larvae, prepupae or adults). An additional model with only prepupae and adults was fitted for *Eo*, confirming the overall higher expression in Controls (regime on poor diet: $F_{1,6} = 12.3$, $P = 0.013$, regime on standard diet: $F_{1,6} = 8.7$, $P = 0.026$).

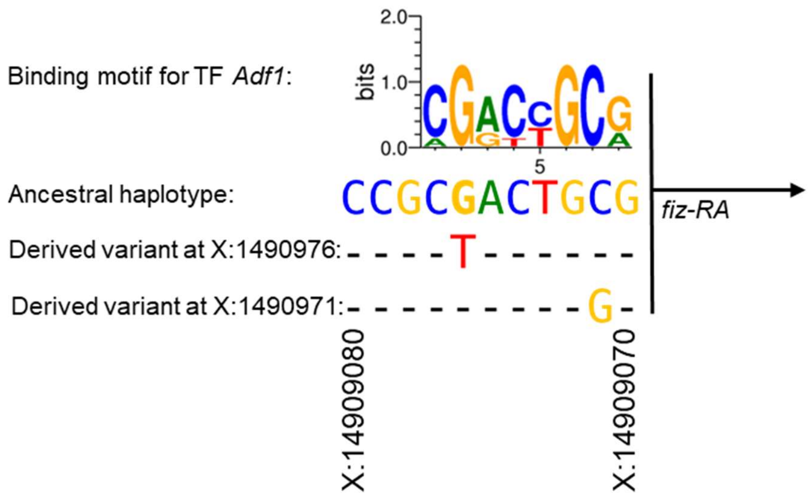

Supplementary Figure C. Alignment of the sequence immediately upstream of the transcription start of *fiz* transcript A with the binding motif of the transcription factor *Adf1*. While the ancestral sequence (associated with low expression) matches the motif ($p = 8 \times 10^{-5}$, $E = 0.11$, $q = 0.026$), the biding affinity is lost by substitution at either position X:14909076 or X:14909071. The motif has been obtained with the *Tomtom* tool of the MEME suite v. 5.5.5.

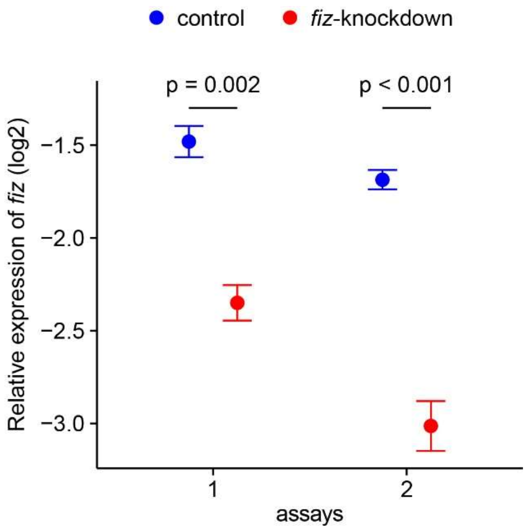

Supplementary Figure D. Relative expression of *fiz* in *fiz*-knockdown and control larvae. Eggs from assay 1 was used for experiment on poor diet; eggs from assay 2 for experiment on standard diet. In both assays, the knockdown was efficient and *fiz* expression was reduced by approximately 40% (assay 1: $F_{1,4} = 46.5$, $P = 0.002$; assay 2: $F_{1,4} = 85.0$, $P < 0.001$). Symbols indicate means ± SE. N = 3 replicates of 10 larvae.

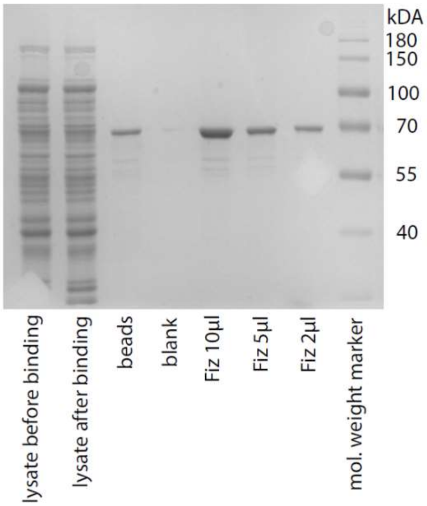

Supplementary Figure E. Coomassie-stained SDS-PAGE gel of Fiz purification. The position of the band corresponds to the expected molecular weight of the protein (70.8 kDa).

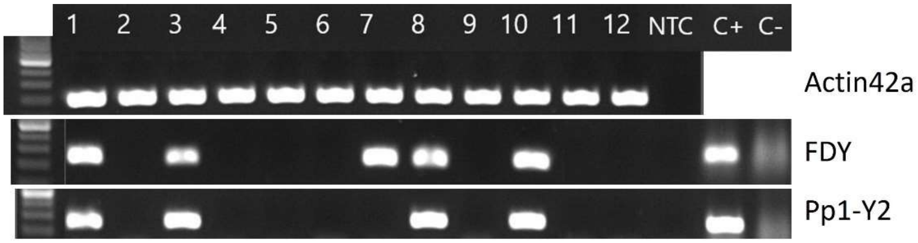

Supplementary Figure F. Verification that samples for allele-specific *fiz* expression consisted exclusively of female larvae (results for the first 12 samples shown). This was based on the absence of PCR amplicon for two Y chromosome genes *FDY* (FBgn0265047) and *Pp1-Y2* (FBgn0046698). C+ and C-: positive and negative controls for *FDY* and *Pp1-Y2*, consisting of DNA pool from a sample six adult females + one adult male (C+) or from seven adult females (C-). NTC: negative control without DNA. Samples with a band for *FDY* and/or *Pp1-Y2* (here samples 1, 3, 7, 8, and 10) must have contained at least one male and were excluded. A summary result is presented in **Table M.**

Tables

Table A. Summary of significance tests from linear mixed models (LMM) for the relative amount of H_2O_2 in samples with purified Fiz + substrate or purified Fiz only or substrate only. Pairwise comparisons are also shown. Fiz = purified Fiz protein; E = ecdysone substrate; 20E = 20-hydroxyecdsone substrate. Results are reported in Figure 2.

Substrate	Factor	Contrast	Estimate	Statistics	*P*
Ecdysone	Type of sample			$F_{2,9}$ = 1431.4	<0.001
		(Fiz + E) – Fiz	32512	t_9 = 40.3	<0.001
		(Fiz + E) – E	40846	t_9 = 50.6	<0.001
		Fiz – E	8334	t_9 = 10.3	<0.001
20E	Type of sample			$F_{2,4}$ = 1537	<0.001
		(Fiz + 20E) – Fiz	22593	t_4 = 38.3	<0.001
		(Fiz + 20E) – 20E	21296	t_4 = 51.0	<0.001
		Fiz – 20E	-1298	t_4 = -2.2	0.18

Table B. Summary of significance tests from LMM of relative expression of *fiz*, *Eo* and *CG9512* in larvae, prepupae and flies, reported in Figure B in S1 Appendix. The reported *P* values for contrasts are after sequential Bonferroni correction for multiple stages.

	Poor diet				Standard diet			
Gene	Factor	Stage contrast CTL – SEL	Statistics	*P*	Factor	Stage contrast CTL – SEL	Statistics	*P*
	Regime		$F_{1,6}$ = 90.6	< 0.001	Regime		$F_{1,4}$ = 340.7	< 0.001
	Stage		$F_{4,54}$ = 5.6	< 0.001	Stage		$F_{5,20}$ = 4.4	0.007
	Regime × Stage		$F_{4,54}$ = 13.4	< 0.001	Regime × Stage		$F_{5,20}$ = 22.9	< 0.001
						Larvae day 3	t_{24} = 4.3	< 0.001
fiz		Larvae day 4	t_{31} = 3.8	0.002		Larvae day 4	t_{24} = 5.9	< 0.001
		Larvae day 5	t_{31} = 3.8	0.002		Larvae day 5	t_{24} = 8.6	< 0.001
		Prepupae	t_{31} = 10.0	< 0.001		Prepupae	t_{24} = 16.8	< 0.001
		Males	t_{31} = 2.6	0.015		Males	t_{24} = 4.9	< 0.001
		Females	t_{31} = 2.9	0.015		Females	t_{24} = 4.9	< 0.001
	Regime		$F_{1,6}$ = 5.7	0.054	Regime		$F_{1,72}$ = 18.7	< 0.001
	Stage		$F_{4,54}$ = 143.9	< 0.001	Stage		$F_{5,72}$ = 72.5	< 0.001
	Regime × Stage		$F_{4,54}$ = 2.1	0.089	Regime × Stage		$F_{5,72}$ = 1.6	0.17
						Larvae day 3	t_{40} = 1.2	0.48
Eo		Larvae day 4	t_{29} = 0.1	0.91		Larvae day 4	t_{40} = 2.2	0.16
		Larvae day 5	t_{29} = 0.9	0.80		Larvae day 5	t_{40} = 0.5	0.62
		Prepupae	t_{29} = 1.2	0.75		Prepupae	t_{40} = 2.6	0.071
		Males	t_{29} = 2.6	0.066		Males	t_{40} = 1.7	0.27
		Females	t_{29} = 2.2	0.16		Females	t_{40} = 2.1	0.19
	Regime		$F_{1,6}$ = 0.2	0.67	Regime		$F_{1,4}$ = 3.4	0.14
	Stage		$F_{4,54}$ = 262.7	< 0.001	Stage		$F_{5,56}$ = 136.2	< 0.001
	Regime × Stage		$F_{4,54}$ = 2.4	0.061	Regime × Stage		$F_{5,56}$ = 6.7	< 0.001
						Larvae day 3	t_{9} = 4.5	0.009
CG9512		Larvae day 4	t_{40} = 2.1	0.22		Larvae day 4	t_{9} = 2.1	0.31
		Larvae day 5	t_{40} = 1.1	1.00		Larvae day 5	t_{9} = 1.2	1.00
		Prepupae	t_{40} = 0.9	1.00		Prepupae	t_{9} = 1.1	1.00
		Males	t_{40} = 0.0	1.00		Males	t_{9} = 0.1	1.00
		Females	t_{40} = 0.7	1.00		Females	t_{9} = 0.2	1.00

Table C. Summary of significance tests from LMM of relative expression of *fiz* between poor and standard diet in prepupae and flies, reported in Figure B in S1 Appendix.

Stage	Factor	Statistics	*P*
	Regime	$F_{1,20} = 521.0$	< 0.001
Prepupae	Diet	$F_{1,20} = 44.9$	< 0.001
	Regime × Diet	$F_{1,20} = 0.1$	0.73
	Regime	$F_{1,4} = 21.0$	0.010
Females	Diet	$F_{1.16} = 20.0$	< 0.001
	Regime × Diet	$F_{1.16} = 0.0$	0.97
	Regime	$F_{1,4} = 7.7$	0.051
Males	Diet	$F_{1.16} = 6.4$	0.022
	Regime × Diet	$F_{1.16} = 0.0$	0.85

Table D. Summary of significance tests from LMM of relative expression of *fiz*, *Eo*, *CG9512*, *CG45065*, *CG9521*, *CG12539*, *Shade*, *Cyp18a1*, *Eip74EF* and *broad* in third instar larvae (synchronized) reported in Figure 3. The reported *P* values for contrasts are after sequential Bonferroni correction for multiple stages.

Gene	Factor	Stage contrast CTL – SEL	Statistics	*P*	Gene	Factor	Stage contrast CTL – SEL	Statistics	*P*
fiz	Regime		$F_{1,6} = 55.9$	< 0.001	*Eo*	Regime		$F_{1,6} = 62.9$	< 0.001
	Stage		$F_{2,48} = 11.2$	< 0.001		Stage		$F_{2,48} = 25.1$	< 0.001
	Regime × Stage		$F_{2,48} = 4.1$	0.023		Regime × Stage		$F_{2,48} = 0.6$	0.53
		Early L3	$t_{12} = 5.3$	< 0.001			Early L3	$t_{18} = 4.7$	< 0.001
		24h- older	$t_{12} = 5.5$	< 0.001			24h- older	$t_{18} = 4.8$	< 0.001
		36h- older	$t_{12} = 6.6$	< 0.001			36h- older	$t_{18} = 3.6$	0.002
CG9512	Regime		$F_{1,6} = 47.9$	< 0.001	*CG45065*	Regime		$F_{1,6} = 1.0$	0.35
	Stage		$F_{2,48} = 8.8$	< 0.001		Stage		$F_{2,48} = 20.9$	< 0.001
	Regime × Stage		$F_{2,48} = 22.0$	< 0.001		Regime × Stage		$F_{2,48} = 1.5$	0.23
		Early L3	$t_{17} = 1.9$	0.069			Early L3	$t_{19} = 0.4$	0.68
		24h- older	$t_{17} = 5.3$	< 0.001			24h- older	$t_{19} = 1.3$	0.59
		36h- older	$t_{17} = 7.0$	< 0.001			36h- older	$t_{19} = 1.0$	0.68
CG9521	Regime		$F_{1,6} = 37.3$	< 0.001	*CG12539*	Regime		$F_{1,6} = 72.3$	< 0.001
	Stage		$F_{2,48} = 56.8$	< 0.001		Stage		$F_{2,48} = 0.1$	0.94
	Regime × Stage		$F_{2,48} = 6.2$	0.004		Regime × Stage		$F_{2,48} = 0.1$	0.92
		Early L3	$t_{18} = 2.3$	0.031			Early L3	$t_{18} = 4.6$	< 0.001
		24h- older	$t_{18} = 4.3$	< 0.001			24h- older	$t_{18} = 5.1$	< 0.001
		36h- older	$t_{18} = 5.6$	< 0.001			36h- older	$t_{18} = 4.9$	< 0.001
Shade	Regime		$F_{1,54} = 2.9$	0.095	*Cyp18a1*	Regime		$F_{1,6} = 3.5$	0.11
	Stage		$F_{2,54} = 21.0$	< 0.001		Stage		$F_{2,48} = 87.2$	< 0.001
	Regime × Stage		$F_{2,54} = 0.0$	0.96		Regime × Stage		$F_{2,48} = 1.8$	0.17
		Early L3	$t_{15} = 0.7$	0.82			Early L3	$t_{16} = 2.0$	0.18
		24h- older	$t_{15} = 1.1$	0.82			24h- older	$t_{16} = 1.4$	0.36
		36h- older	$t_{15} = 0.9$	0.82			36h- older	$t_{16} = 0.5$	0.60
Eip74EF	Regime		$F_{1,6} = 0.8$	0.39	*broad*	Regime		$F_{1,54} = 0.6$	0.44
	Stage		$F_{2,48} = 60.9$	< 0.001		Stage		$F_{2,54} = 279.8$	< 0.001
	Regime × Stage		$F_{2,48} = 1.0$	0.39		Regime × Stage		$F_{2,54} = 2.0$	0.15
		Early L3	$t_{18} = 0.1$	1.00			Early L3	$t_{15} = 1.9$	0.23
		24h- older	$t_{18} = 1.4$	0.50			24h- older	$t_{15} = 0.6$	1.00
		36h- older	$t_{18} = 0.3$	1.00			36h- older	$t_{15} = 0.0$	1.00

Table E. Sequences of PCR primers used in the study. Primers used in amplicon sequencing include overhangs (in color).

Gene	Direction	Sequence 5' -> 3'	Use	Source
αTub84B	forward	TGTCGCGTGTGAAACACTTC	RT-qPCR	Ponton et al. 2011
	reverse	AGCAGGCGTTTCCAATCTG	RT-qPCR	
eEF1α2	forward	GCGTGGGTTTGTGATCAGTT	RT-qPCR	Ponton et al. 2011
	reverse	GATCTTCTCCTTGCCCATCC	RT-qPCR	
RpL32	forward	ATGCTAAGCTGTCGCACAAATG	RT-qPCR	Ponton et al. 2011
	reverse	GTTCGATCCGTAACCGATGT	RT-qPCR	
fiz	forward	TCTGAGTTGCCGGCACTATT	RT-qPCR	designed by authors
	reverse	CATTCACTCCTCCCGATCCT	RT-qPCR	
Eo	forward	GAGCTGTGCCAGGTGAAGG	RT-qPCR	www.flyrnai.org/flyprimerbank
	reverse	GGTCAGACCAAAGATTTCCGATT	RT-qPCR	
CG9512	forward	GCCGAAAGTGTGACCTTTGT	RT-qPCR	designed by authors
	reverse	ATGCCTGAAAGCAACAGGAT	RT-qPCR	
CG45065	forward	GGAGCCAACCGTCATACGTC	RT-qPCR	designed by authors
	reverse	TCCGAGATCTCCGTTTCATC	RT-qPCR	
CG9521	forward	ATGGCAAGCAAATCGCTGATA	RT-qPCR	www.flyrnai.org/flyprimerbank
	reverse	CGTCTCGAAAAGGACGTTATTGT	RT-qPCR	
CG12539	forward	AGCTCAACCAGGTGGGATT	RT-qPCR	designed by authors
	reverse	GCTCCAGCTCCAATCACAAT	RT-qPCR	
shade	forward	CGCTTAATGCAGGGACTGTG	RT-qPCR	designed by authors
	reverse	GCTCTGGGGTAAACTGCTTG	RT-qPCR	
Cyp18a1	forward	GCTTCGATCCCAACAACATT	RT-qPCR	designed by authors
	reverse	TGTACCAGTTGCTCCTCGTG	RT-qPCR	
broad	forward	ACAACAACAGCCCCGACTT	RT-qPCR	designed by authors
	reverse	CGTTGCGCTTCTCCTCCTT	RT-qPCR	
Eip74EF	forward	GCTGCTCCACAATCTGCTTAG	RT-qPCR	designed by authors
	reverse	GCGGAAATGAACCTGTTGTG	RT-qPCR	
Pp1-Y2	forward	TGAGTCGGCTGGAATTAACC	PCR	designed by authors
	reverse	TGTCAGGAACGTCACATGGT	PCR	
FDY	forward	TTGCAAACTCGTGTGTGTTC	PCR	designed by authors
	reverse	GTTTGCCTAAGTTAAAGTATTGGATT	PCR	
Act42A	forward	CAGATGTGGATCTCGAAGCA	PCR	designed by authors
	reverse	TTCTGAAGGAGCGGAAGTGT	PCR	
fiz	forward	TCGTCGGCAGCGTCAGATGTGTATAAGAGACAG CTGCAGCACACGAACTTCAC	Amplicon sequencing	designed by authors
	reverse	GTCTCGTGGGCTCGGAGATGTGTATAAGAGACAG AGCAGCGACCATCTTTCATT	Amplicon sequencing	
CG9512	forward	TCGTCGGCAGCGTCAGATGTGTATAAGAGACAG CGTAACAATCGAGCCGAAAG	Amplicon sequencing	designed by authors
	reverse	GTCTCGTGGGCTCGGAGATGTGTATAAGAGACAG CGGACACGATGACCTCCTTA	Amplicon sequencing	

Table F. Summary of significance tests from generalized linear mixed models (GLMM) for allele specific expression of *fiz* and *CG9512* in females reported in Figure 4A and 4B. F1 = average of F1 from both crosses (CTL × SEL and SEL × CTL); Mix = mix parental lines. Custom contrasts "F1" and "Mix" are compared to an allele frequency of 50%. In custom contrast "(CTL × SEL) – (SEL × CTL)", allele frequency of CTL × SEL is compared to the one of SEL × CTL; "F1 – Mix", allele frequency of all F1 (both direction of crosses) is compared to the allele frequency in mix parent lines.

Gene	Custom Contrasts	Estimate	Statistics	*P*
fiz	F1	-1.73	$t_4 = 31.8$	< 0.001
	Mix	-2.11	$t_6 = 26.6$	< 0.001
	(CTL × SEL) – (SEL × CTL)	-0.06	$t_4 = 0.6$	0.61
	F1 – Mix	0.37	$t_4 = 3.9$	0.017
CG9512	F1	-0.13	$t_4 = 1.2$	0.30
	Mix	-1.10	$t_6 = 7.4$	< 0.001
	(CTL × SEL) – (SEL × CTL)	0.01	$t_4 = 0.1$	0.95
	F1 – Mix	0.97	$t_4 = 5.5$	0.005

Table G. Summary of significance tests from LMM for *fiz* and *CG9512* expression in males reported in Figure 4C-D.

Gene	Pairwise comparisons	Estimate	Statistics	*P*
fiz	Control – (CTL × SEL)	0.12	$t_{18} = 0.3$	0.99
	Control – (SEL × CTL)	2.48	$t_{18} = 7.0$	< 0.001
	Control – Selected	3.23	$t_{18} = 9.1$	< 0.001
	(CTL × SEL) – (SEL × CTL)	2.36	$t_{18} = 6.6$	< 0.001
	(CTL × SEL) – Selected	3.11	$t_{18} = 8.8$	< 0.001
	(SEL × CTL) – Selected	0.75	$t_{18} = 2.1$	0.19
CG9512	Control – (CTL × SEL)	0.19	$t_{18} = 1.3$	0.60
	Control – (SEL × CTL)	1.13	$t_{18} = 7.3$	< 0.001
	Control – Selected	1.36	$t_{18} = 8.8$	< 0.001
	(CTL × SEL) – (SEL × CTL)	0.93	$t_{18} = 6.1$	<0.001
	(CTL × SEL) – Selected	1.17	$t_{18} = 7.6$	<0.001
	(SEL × CTL) – Selected	0.24	$t_{18} = 1.5$	0.44

Table H. Summary of significance tests from LMM for inverse of developmental time. Results are reported in Figure 5B. Genotype: control *versus fiz* knockdown.

Diet	Factor	Contrasts control – *fiz* knockdown	Statistics	*P*
Poor	Genotype		$F_{1,6} = 18.9$	0.005
	Sex		$F_{1,6} = 1.1$	0.33
	Genotype × Sex		$F_{1,6} = 4.5$	0.077
		in Females	$t_9 = 4.8$	< 0.001
		in Males	$t_9 = 2.9$	0.019
Standard	Genotype		$F_{1,8} = 0.3$	0.59
	Sex		$F_{1,8} = 96.0$	< 0.001
	Genotype × Sex		$F_{1,8} = 0.1$	0.76
		in Females	$t_8 = 0.5$	0.61
		in Males	$t_8 = 0.6$	0.56

Table I. Summary of significance tests from LMM for female weight. Pairwise comparisons were done from the model with developmental time as a factor and without quadratic factors (see Methods). Results are reported in Figure 5C. Genotype: control *versus fiz* knockdown; Dev time: developmental time. The reported *P* values for contrasts are after sequential Bonferroni correction for multiple days.

Diet	Factor	Contrasts control – fiz knockdown	Statistics	*P*
Poor	Genotype		$F_{1,10} = 25.1$	< 0.001
	Dev time		$F_{1,35} = 10.2$	0.003
	(Dev time)^2		$F_{1,35} = 38.5$	< 0.001
	Genotype × Dev time		$F_{1,35} = 0.7$	0.42
	Genotype × (Dev time)^2		$F_{1,35} = 2.8$	0.10
		Day 15	$t_{32} = 4.1$	0.002
		Day 16	$t_{25} = 5.3$	< 0.001
		Day 17	$t_{27} = 4.1$	0.002
		Day 18	$t_{25} = 4.1$	0.002
		Day 19	$t_{25} = 3.4$	0.005
		Day 20	$t_{27} = 3.2$	0.005
Standard	Genotype		$F_{1,20} = 13.0$	0.001
	Dev time		$F_{1,14} = 39.3$	< 0.001
	(Dev time)^2		$F_{1,14} = 0.9$	0.35
	Genotype × Dev time		$F_{1,14} = 4.1$	0.062
	Genotype × (Dev time)^2		$F_{1,14} = 0.2$	0.70
		Day 12	$t_{21} = -4.1$	0.002
		Day 13	$t_{21} = -3.7$	0.003
		Day 14	$t_{21} = -1.9$	0.070

Table J. Summary of significance tests from LMM for female growth rate. Pairwise comparisons were done from the model with developmental time as a factor and without quadratic factors (see methods). Results are reported in Figure 5D. Genotype: control *versus fiz* knockdown; Dev time: developmental time. The reported *P* values for contrasts are after sequential Bonferroni correction for multiple days.

Diet	Factor	Contrasts control – fiz knockdown	Statistics	*P*
Poor	Genotype		$F_{1,12} = 27.4$	< 0.001
	Dev time		$F_{1,36} = 3318.2$	< 0.001
	(Dev time)^2		$F_{1,35} = 192.6$	< 0.001
	Genotype × Dev time		$F_{1,36} = 2.1$	0.15
	Genotype × (Dev time)^2		$F_{1,35} = 4.0$	0.052
		Day 15	$t_{31} = 5.0$	< 0.001
		Day 16	$t_{24} = 6.9$	< 0.001
		Day 17	$t_{27} = 5.1$	< 0.001
		Day 18	$t_{24} = 4.8$	< 0.001
		Day 19	$t_{24} = 3.6$	0.003
		Day 20	$t_{27} = 3.0$	0.006
Standard	Genotype		$F_{1,20} = 15.6$	< 0.001
	Dev time		$F_{1,14} = 4472.9$	< 0.001
	(Dev time)^2		$F_{1,14} = 38.0$	< 0.001
	Genotype × Dev time		$F_{1,14} = 9.6$	0.008
	Genotype × (Dev time)^2		$F_{1,14} = 0.0$	0.85
		Day 12	$t_{22} = 5.3$	< 0.001
		Day 13	$t_{21} = 4.1$	0.001
		Day 14	$t_{21} = 1.8$	0.091

Table K. Comparison of the effect of *fiz*-knockdown on performance traits of flies raised on poor *versus* standard diet. The absolute estimates and standard errors are reported for each trait. *P*: p-value from t-test comparing the absolute estimate on poor *versus* standard diet.

trait	sex	Poor diet		Standard diet		*P*
		Absolute estimate	Standard error	Absolute estimate	Standard error	
Survival	both	7.76e-01	1.07e-01	5.98e-01	1.10e-01	0.156
Developmental time	female	3.41e-03	7.05e-04	7.79e-04	1.48e-03	0.002
	male	2.02e-03	7.05e-04	8.93e-04	1.48e-03	0.002
Weight	female	3.73e-02	7.44e-03	2.81e-02	7.77e-03	0.011
Growth rate	female	1.32e-02	2.53e-03	1.02e-02	2.59e-03	0.004

Table L. Raw data on abundance of FAD (peak area; arbitrary units) in samples containing purified Fiz with a substrate (ecdysone or 20E) or only the substrate. n.d. not detected. The presence of FAD in samples containing Fiz suggests that Fiz, predicted to be a flavoprotein, is properly folded.

Samples	Presence of Fiz	Substrate	FAD (peak area)
Fiz_1	Yes	Ecdysone	65905
Fiz_2	Yes	Ecdysone	99808
Fiz_3	Yes	Ecdysone	73717
Fiz_4	Yes	Ecdysone	77037
Fiz_5	Yes	20E	97036
Fiz_6	Yes	20E	103759
Fiz_7	Yes	20E	97977
Fiz_8	Yes	20E	93297
N_1	No	Ecdysone	518
N_2	No	Ecdysone	698
N_3	No	Ecdysone	n.d.
N_4	No	Ecdysone	239
N_5	No	20E	n.d.
N_6	No	20E	674
N_7	No	20E	918
N_8	No	20E	693

Table M. Summary of the verification that samples for allele-specific *fiz* expression consists exclusively of female larvae. Verification is based on PCR targetting two genes located on the Y-chromosome: *FDY* (FBgn0265047) and *Pp1-Y2* (FBgn0046698). A “yes” or “no” indicate a presence or absence, respectively, of a band for the corresponding gene. See Figure F for more details and the gel image corresponding to the first 12 samples. A gray background indicates a discarded sample (i.e. with at least one male larva). The last column indicates samples of Selected and Control populations used to generate the 50:50 mixed samples of cDNA reported in Figure 4A and B.

Sample	Population	Type	*FDY*	*Pp1-Y2*	Decision	cDNA_mixed
1	C2	Control	Yes	Yes	Discard	
2	C4	Control	No	No	Keep	Mix_B1
3	C6	Control	Yes	Yes	Discard	
4	S1	Selected	No	No	Keep	
5	S2	Selected	No	No	Keep	Mix_B1
6	S3	Selected	No	No	Keep	
7	C2×S1	CTL×SEL	Yes	No	Discard	
8	C4×S2	CTL×SEL	Yes	Yes	Discard	
9	C6×S3	CTL×SEL	No	No	Keep	
10	S1×C2	SEL×CTL	Yes	Yes	Discard	
11	S2×C4	SEL×CTL	No	No	Keep	
12	S3×C6	SEL×CTL	No	No	Keep	
13	C2	Control	No	No	Keep	Mix_A1
14	C4	Control	No	No	Keep	Mix_B2
15	C6	Control	No	No	Keep	Mix_C1
16	S1	Selected	No	No	Keep	Mix_A1
17	S2	Selected	No	No	Keep	Mix_B2
18	S3	Selected	No	No	Keep	Mix_C1
19	C2×S1	CTL×SEL	No	No	Keep	
20	C4×S2	CTL×SEL	No	No	Keep	
21	C6×S3	CTL×SEL	No	No	Keep	
22	S1×C2	SEL×CTL	No	No	Keep	
23	S2×C4	SEL×CTL	No	No	Keep	
24	S3×C6	SEL×CTL	No	No	Keep	
25	C2	Control	No	No	Keep	Mix_A2
26	C4	Control	Yes	Yes	Discard	
27	C6	Control	No	No	Keep	Mix_C2
28	S1	Selected	No	No	Keep	Mix_A2
29	S2	Selected	No	No	Keep	
30	S3	Selected	No	No	Keep	Mix_C2
31	C2×S1	CTL×SEL	No	No	Keep	
32	C4×S2	CTL×SEL	No	No	Keep	
33	C6×S3	CTL×SEL	No	No	Keep	
34	S1×C2	SEL×CTL	No	No	Keep	
35	S2×C4	SEL×CTL	No	No	Keep	
36	S3×C6	SEL×CTL	No	No	Keep	
37	C2	Control	Yes	Yes	Discard	
38	C4	Control	Yes	Yes	Discard	
39	C6	Control	No	No	Keep	Mix_C3
40	S1	Selected	No	No	Keep	
41	S2	Selected	No	No	Keep	
42	S3	Selected	No	No	Keep	Mix_C3
43	C4×S2	CTL×SEL	No	No	Keep	
44	C6×S3	CTL×SEL	No	No	Keep	
45	S1×C2	SEL×CTL	No	No	Keep	
46	S2×C4	SEL×CTL	No	No	Keep	
47	S3×C6	SEL×CTL	No	No	Keep	
